# Identification and characterization of a novel *DGAT1* missense mutation associated with congenital diarrhea[S]

**DOI:** 10.1194/jlr.P075119

**Published:** 2017-04-03

**Authors:** Nina L. Gluchowski, Chandramohan Chitraju, Joseph A. Picoraro, Niklas Mejhert, Shirly Pinto, Winnie Xin, Daniel S. Kamin, Harland S. Winter, Wendy K. Chung, Tobias C. Walther, Robert V. Farese

**Affiliations:** Division of Gastroenterology and Nutrition,* Boston Children’s Hospital, Boston, MA 02115; Department of Genetics and Complex Diseases,† Harvard T. H. Chan School of Public Health, Boston, MA 02115; Harvard Medical School,§ Boston, MA 02115; Division of Pediatric Gastroenterology, Hepatology, and Nutrition,** Columbia University Medical Center, New York, NY 10027; Merck & Co., Inc.,†† Kenilworth, NJ 07033; Department of Neurology,§§ Massachusetts General Hospital, Boston, MA 02114; Division of Gastroenterology,*** MassGeneral Hospital for Children, Boston, MA 02114; Departments of Pediatrics and Medicine,††† Columbia University Medical Center, New York, NY 10027; Broad Institute of Harvard and Massachusetts Institute of Technology,§§§ Cambridge, MA 02142; Howard Hughes Medical Institute,**** Boston, MA 02115

**Keywords:** protein-losing enteropathy, intestine, genetics, triglycerides, lipid droplets, diet and dietary lipids, acyl CoA:diacylglycerol acyltransferase

## Abstract

Acyl-CoA:diacylglycerol acyltransferase (DGAT)1 and DGAT2 catalyze triglyceride (TG) biosynthesis in humans. Biallelic loss-of-function mutations in human *DGAT1* result in severe congenital diarrhea and protein-losing enteropathy. Additionally, pharmacologic inhibition of DGAT1 led to dose-related diarrhea in human clinical trials. Here we identify a previously unknown *DGAT1* mutation in identical twins of South Asian descent. These male patients developed watery diarrhea shortly after birth, with protein-losing enteropathy and failure to thrive. Exome sequencing revealed a homozygous recessive mutation in *DGAT1*, c.314T>C, p.L105P. We show here that the p.L105P DGAT1 enzyme produced from the mutant allele is less abundant, resulting in partial loss of TG synthesis activity and decreased formation of lipid droplets in patient-derived primary dermal fibroblasts. Thus, in contrast with complete loss-of-function alleles of *DGAT1*, the p.L105P missense allele partially reduces TG synthesis activity and causes a less severe clinical phenotype. Our findings add to the growing recognition of DGAT1 deficiency as a cause of congenital diarrhea with protein-losing enteropathy and indicate that *DGAT1* mutations result in a spectrum of diseases.

Acyl-CoA:diacylglycerol acyltransferase (DGAT)1 and DGAT2 catalyze the final step in triglyceride (TG) biosynthesis (1, 2), and these two enzymes account for nearly all TG synthesis in mammals (3, 4). TGs and other neutral lipids are stored in cells in cytosolic lipid droplets (5–7). The processes of TG synthesis and storage are central to energy metabolism and metabolic processes, such as intestinal fat absorption (8), liver TG storage and secretion (9), cardiac metabolism (10, 11), and TG storage in adipocytes (12) and macrophages of adipose tissue (13, 14).

Complete loss of DGAT1 function yields different phenotypes in mice and humans. DGAT1-null mice have a favorable phenotype that includes decreased adiposity, increased energy expenditure, resistance to diet-induced obesity and fatty liver, increased insulin sensitivity, and increased longevity (3, 15, 16). These favorable metabolic effects motivated the development of numerous DGAT1 inhibitors as therapeutic agents to treat hypertriglyceridemia or other obesity-related diseases (17). However, administration of DGAT1 inhibitors in humans resulted in dose-related gastrointestinal side effects, most notably diarrhea (17).

Concomitantly, we reported that humans with homozygous complete loss-of-function mutations in *DGAT1* have a severe congenital diarrhea syndrome (18). The first reported mutation, chromosome 8 145541756 A→G, was found in the Ashkenazi Jewish population and causes exon 8 to be skipped in the expressed protein. The mutant protein is unstable, thus resulting in a complete loss of DGAT1 activity. Patients with this mutation develop severe diarrhea in the neonatal period, with protein-losing enteropathy and associated hypoalbuminemia and hypertriglyceridemia. These children required periods of parenteral nutrition and intralipid supplementation. One child described in the initial report died from complications of malnutrition and sepsis. More recently, a missense mutation in *DGAT1*, p.L295P, was reported (19). This mutation is associated with a similar clinical syndrome, decreased *DGAT1* mRNA and protein levels in dermal fibroblasts from patients when compared with control fibroblasts, presumably resulting in decreased TG synthesis activity, although this was not directly tested (19). Taken together, these findings indicate that mice tolerate complete DGAT1 loss relatively well, but humans with complete genetic deficiency or pharmacological inactivation of DGAT1 are sensitive to decreased DGAT1 activity resulting in moderate to severe diarrhea.

In the current study, we identify and characterize a previously unknown *DGAT1* missense mutation that results in a partial loss of function and a less severe clinical phenotype, expanding the phenotypic spectrum of the human disease. We also discuss the therapy for DGAT1-deficient patients and describe dietary modification as a successful treatment option.

## EXPERIMENTAL PROCEDURES

### Mutation identification

Genomic DNA was extracted from whole blood of monochorionic twins and their parents. Exome sequencing was performed as described (20). Briefly, the exome was captured with an Agilent SureSelect XT2 All Exon V4 kit (Agilent Technologies, Santa Clara, CA) and sequenced by IlluminaHiSeq 2000 platform (Illumina, San Diego, CA). Reads were aligned to the human reference genome UCSC hg19/GRCh37 (hgdownload.cse.ucsc.edu), and known protein-coding RefSeq genes (ncbi.nlm.nih.gov/refseq/). Local realignment around insertion-deletion sites was performed with the Genome Analysis Toolkit (software.broadinstitute.org/gatk/) and variant calls were generated using SAMtools (htslib.org). Variants were annotated by allele frequency using 1000 Genomes (http://www.internationalgenome.org), National Heart, Lung, and Blood Institute GO Exome Sequencing Project (esp.gs.washington.edu/drupal/), Exome Aggregation Consortium (exac.broadinstitute.org), and the Genome Aggregation Database (gnomad.broadinstitute.org), and by functional predictions using PolyPhen-2 (genetics.bwh.harvard.edu/pph2/), sorting intolerant from tolerant (sift.jcvi.org), genome evolutionary rate profiling (mendel.stanford.edu/SidowLab/downloads/gerp/), and combined annotation-dependent depletion (cadd.gs.washington.edu). Variants with minor allele frequency greater than 1% were filtered out and the remaining variants were separated based on predicted inheritance pattern, including de novo changes. Candidate genes were prioritized based on functional predictions and phenotypic correlation from Human Gene Mutation Database (http://www.hgmd.cf.ac.uk/ac/index.php), National Heart, Lung, and Blood Institute Exome Variant Server (evs.gs.washington.edu/EVS/), Online Mendelian Inheritance of Man (omim.org), PubMed (ncbi.nlm.nih.gov/pubmed/), and ClinVar (ncbi.nlm.nih.gov/clinvar/). Whole-exome sequencing was performed on DNA from unaffected siblings, as described (18).

The *DGAT1* genetic results for the patients and their parents were confirmed by Sanger sequencing. Exon 3 of the *DGAT1* gene was amplified using the primer pair: forward 5′-AGAGGTGTTGGGAGGATCTG-3′ and reverse 5′-GTCTGAGTGGGTGGCAGGT-3′. PCR amplification was carried out using *Taq* DNA polymerase (Qiagen, Valencia, CA) and a standard PCR profile. PCR products were purified using the QIAquick Multiwell PCR purification system (Qiagen) and the purified products were sequenced bi-directionally by being labeled with Big Dye Terminator (Applied Biosystems of Thermo Fisher Scientific, Waltham, MA) and resolved by capillary electrophoresis on an ABI 3500xL genetic analyzer (Applied Biosystems). Positive mutations were identified by comparing bi-directional sequence data to a normal reference sequence.

### Cell lines

Primary dermal fibroblasts from both patients were provided by the Manton Center for Orphan Disease Research at Boston Children’s Hospital. Primary dermal fibroblasts from three control subjects were provided by the Memory and Aging Center at the University of California, San Francisco; the control cell lines used were CTR2 and V, as described (21, 22), and JJ (unpublished). Primary human dermal fibroblasts were cultured in Ham’s F-10 nutrient mix (Thermo Fisher Scientific) containing FBS (10%), penicillin (100 U/ml), and streptomycin (100 mg/ml).

Human breast cancer cells (SUM159 cells) were maintained in DMEM/F-12 GlutaMAX (Life Technologies, Carlsbad, CA) containing FBS (5%), penicillin (100 U/ml), streptomycin (100 mg/ml), hydrocortisone (1 mg/ml) (Sigma-Aldrich, St. Louis, MO), insulin (5 mg/ml) (Cell Applications, San Diego, CA), and HEPES (10 mM; pH 7.0). Transfection of plasmid was performed using Lipofectamine 3000 (Life Technologies) according to the manufacturer’s instructions.

### DGAT inhibitors

DGAT1 and DGAT2 inhibitors were from Merck & Co., Inc., Kenilworth, NJ (23, 24). The inhibitors were suspended in DMSO and used at a final concentration of 5 μM.

### Antibodies

Rabbit polyclonal antibody against human DGAT1 was custom generated (GenScript, Piscataway, NJ), affinity purified, and used at a 1:1,000 dilution. The peptide sequence used for the epitope was “GGGGPAAAEEEVRDAAAGPDVGAAGDAPAP” and correlates to amino acids 20–49 of human DGAT1. Other antibodies were anti-flag M2 monoclonal antibody (1:1,000) produced in mouse (Sigma-Aldrich) and calnexin polyclonal antibody SPA-860 (1:1,000) produced in rabbit (Enzo Life Sciences, Farmingdale, NY). Secondary antibodies were anti-mouse IgG-HRP (1:10,000) and anti-rabbit IgG-HRP (1:10,000) (Santa Cruz Biotechnology, Dallas, TX), and anti-rabbit IgG-HRP (1:5,000) (GenScript).

### cDNA and plasmids

Wild-type *DGAT1* with a FLAG tag sequence in a modified pMSCV vector with a CMV promoter was used as described (18). *DGAT1* c.314C>T with a FLAG tag sequence was synthesized as a gBlock (Integrated DNA Technologies, Coralville, IA), PCR-amplified, and cloned into the same modified pMSCV vector as the wild-type.

### RNA extraction and quantitative real-time PCR

Total RNA from human dermal fibroblasts was isolated using the RNeasy kit (Qiagen) according to the manufacturer’s instructions. cDNA was synthesized using iScript cDNA synthesis kit (Bio-Rad), and quantitative real-time PCR was performed in triplicates using SYBR Green PCR Master Mix kit (Applied Biosystems).

### Immunoblotting

Cells were lysed in buffer containing sucrose (250 mM), Tris-HCl (pH 7.4) (50 mM), and cOmplete Mini EDTA-free protease inhibitor cocktail (Sigma-Aldrich), and treated with sonication at 30 kHz for 1 s 14 times. Protein lysates were separated on a 4–15% SDS-PAGE gel (BioRad, Hercules, CA) and transferred to a PVDF membrane (BioRad). The membrane was blocked with TBST containing 5% milk for 1 h and incubated with primary antibodies at a 1:1,000 dilution at 4°C overnight. The membrane was washed with TBST for 5 min three times and then incubated with secondary antibody at a 1:5,000 or 1:10,000 dilution at room temperature for 1 h. The membrane was washed again with TBST for 5 min three times and revealed using SuperSignal West Pico chemiluminescent substrate kit (Thermo Fisher Scientific) per the manufacturer’s instructions.

### Protein degradation assay

SUM159 cells were treated with cycloheximide (50 μM in culture medium) 24 h after transfection. Cells were lysed at different time points as indicated, and immunoblots were performed using anti-FLAG primary antibody as described above.

### DGAT1 activity assays

DGAT1 activity was measured in vitro with assay conditions that were selective for DGAT1 (2). Total cell lysate (10 μg protein) was incubated with DGAT inhibitors (5 μM) for 30 min where indicated. The lysate was then added to a reaction mixture of Tris-HCl (pH 7.5) (100 mM), MgCl_2_ (25 mM), BSA (0.625 mg/ml), oleoyl-CoA (25 μM) that contained [^14^C]oleoyl-CoA as a tracer, and 1,2 diacylglycerol (200 μM) in a final volume of 200 μl. Reactions were carried out at 37°C for 1–20 min as indicated. The reactions were stopped by adding chloroform:methanol (2:1). After stopping the reaction, lipids were extracted by adding phosphoric acid (2%) and centrifugation. The lipids were dried, resuspended in chloroform, and separated by TLC with a hexane:diethyl ether:acetic acid (80:20:1) solvent system. TLC plates were exposed to phosphor imaging cassette for 48 h and revealed by Typhoon FLA 7000 phosphor imager (GE Healthcare Life Sciences, Pittsburgh, PA). Lipids on the TLC plate were stained with iodine vapors; TG bands were scraped and quantified by liquid scintillation counter.

To measure TG synthesis in cells, cells were pretreated with DGAT inhibitor (5 μM in culture medium) as indicated for 30 min, and then treated with oleate (400 μM in culture medium) and DGAT inhibitor (5 μM in culture medium) as indicated for 4 h. Lipids were extracted directly from 6-well culture plates by adding hexane:isopropanol 3:2 with gentle shaking for 10 min. These lipids were separated on TLC plates and quantified as above. Proteins were extracted with 0.3 N NaOH and 0.1% SDS. The TG synthesis results were normalized for total protein in each sample.

### Microscopy

Cells were grown on glass bottom dishes and treated with oleate (400 μM in culture medium) for 4 h. BODIPY 493/503 (1 μl/1 ml) (Thermo Fisher Scientific) was added to stain for neutral lipids just prior to imaging. Images were obtained with a spinning disk confocal (Yokogawa CSU-X1) set up on a Nikon Eclipse Ti inverted microscope with a 60× ApoTIRF 1.4 NA objective (Nikon, Melville, NY). Images were processed manually using FIJI software (fiji.sc), and lipid droplet number was quantified using CellProfiler (Cellprofiler.org).

### Study approval and consent

The Institutional Review Board at Columbia University Medical Center and Massachusetts General Hospital approved this study; the genetics were performed at these institutions. The Institutional Review Board at Boston Children’s Hospital approved this research (Institutional Review Board protocol number 10-02-0053, “Manton Center for Orphan Disease Research Gene Discovery Core”). Written informed consent was obtained from adult participants and from the parents of the children.

### Statistical analysis

Data are presented as means ± standard deviations. Statistical significance was evaluated with Wilcoxon rank sum test using IBM SPSS Statistics for Windows version 23.0 (IBM Corp., Armonk, NY) where indicated, and linear regression using GraphPad Prism for Mac OS X version 7.0a (GraphPad Software, La Jolla, CA) where indicated.

## RESULTS

### Case report

Monochorionic male twins were born at 36 weeks gestational age by scheduled Caesarean section to parents of South Asian descent. Shortly after birth, the twins developed watery diarrhea and failure to thrive that persisted despite multiple formula changes. Evaluations for cystic fibrosis, immune deficiencies, and mitochondrial disorders were nondiagnostic. Hematoxylin and eosin-stained sections of gastric, duodenal, and colonic biopsies at 19 months of age were normal (data not shown). Twin A presented for hospital admission at 26 months with severe failure to thrive. He was receiving gastrostomy tube feeds with a hydrolyzed formula to provide 118 kcal/kg/day. He had six to seven episodes of watery nonbloody diarrhea daily. His weight was 7 kg (z-score −6.14) and height was 74 cm (z-score −4.12). Serum analyses were notable for metabolic acidosis, elevated transaminases, low serum albumin, low total protein and immunoglobulin G levels, iron deficiency anemia, vitamin D and E deficiencies, low zinc and copper levels, and hypertriglyceridemia with serum TG level 112 mg/dl (normal for age is <75 mg/dl). An essential fatty acid profile showed low linoleic acid of 1,429 nmol/ml (normal range 1,600–3,500 nmol/ml) and normal levels of linolenic acid, triene:tetraene ratio, and mead acid. Stool studies showed elevated α-1 antitrypsin, normal 24 h fecal fat, normal elastase, and were negative for bacteria, viruses, and parasites. The patient’s growth and abnormal laboratory values improved with total parenteral nutrition and intralipid supplementation without enteral feeds. He received red blood cell transfusion and intravenous immune globulin. Subsequently, he was successfully transitioned to a diet containing no more than 10% calories from fat and was weaned off parenteral nutritional support. Selected growth and laboratory parameters for twin A are displayed in **Table 1**.

**TABLE 1.** Laboratory and clinical parameters for twin A at different times

	Age (months)		
	26	28	31
Laboratory value (normal range)			
Stool α-1 antitrypsin (0–0.5 mg/g)	>1.13 (H)	0.08	0.23
Albumin (3–4.6 g/dl)	3.0	4.2	4.3
IgG (400–1,300 mg/dl)	109 (L)	603	509
Hemoglobin (11–12.8 g/dl)	7.4 (L)	12.2	12.6
Iron (50–120 mg/dl)	13 (L)		72
25-OH vitamin D (30–80 ng/ml)	12.5 (L)	44.9	41.9
TG (<75 mg/dl)	112 (H)		56
Growth parameter			
Weight (kg)	7.05	8.62	9.63
Weight z-score	−6.14	−4.27	−3.47
Height (cm)	74	76	83
Height z-score	−4.12	−3.97	−2.1

Laboratory results and growth parameters for twin A at 26 months of age while on an extensively hydrolyzed formula, at 28 months of age status post parenteral nutrition and on a low-fat enteral diet, and at 31 months of age continuing on a low-fat diet. Growth parameter z-scores based on CDC growth chart for boys 2–20 years old. H, high; L, low.

**Fig. 1.**
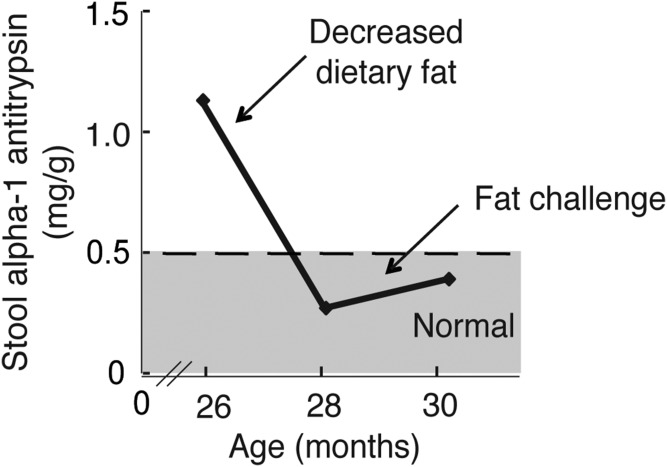
The protein-losing enteropathy associated with DGAT1 deficiency correlates with levels of dietary fat. Stool α-1 antitrypsin levels, a possible marker of disease activity, correlates with varying amounts of dietary fat over time for twin B.

Twin B, for unclear reasons, was not as severely affected. Twin A experienced weight loss during a viral gastroenteritis 2 months prior to his admission, which might have contributed to the more severe symptoms. Twin B did not require hospitalization or parenteral nutrition, but he did exhibit protein-losing enteropathy with elevated stool α-1 antitrypsin levels and loose stools. His stooling pattern, growth, and laboratory serum test values improved on a very low fat enteral diet. **Figure 1** shows the amount of α-1 antitrypsin in the stool for twin B over time as it related to the amount of fat in the diet.

### Identification of homozygous *DGAT1* p.L105P mutation in affected patients

Exome sequencing of the monochorionic twins and their parents revealed that the twins were monozygous and were homozygous for two autosomal variants: *ZNF517*, c.1342G>A; p.G448S and *DGAT1*, c.314C>T, p.L105P. ZNF517 encodes a C2H2-zinc finger protein that is expressed mainly in the brain, testis, prostate, thyroid, and thymus, with relatively low expression levels in the small intestine (25), and missense mutations of *ZNF517* have not been linked to the observed disease phenotype (ClinVar, ncbi.nlm.nih.gov/clinvar/). Because *DGAT1* mutations cause a congenital diarrhea syndrome (18, 19), we focused further characterization on this variant. In addition to the parents, one of two sisters was found to be a heterozygous carrier for the *DGAT1* mutation, while the other sister was homozygous for the wild-type allele. The c.314C>T allele is rare: analysis of the current Genome Aggregation Database (gnomad.broadinstitute.org) revealed that three individuals carry this allele out of 165,092 examined alleles and no homozygotes have been reported. The ExAc database (exac.broadinstitute.org) showed one carrier of 23,030 examined alleles and no homozygotes. The few individuals that carry the *DGAT1* p.L105P mutation in these databases are of the South Asian population. Analysis of the patient haplotypes indicated that this mutation likely arose in common founder in the parents’ population of origin. Inasmuch as the patients’ clinical syndrome matched that previously reported for human DGAT1 deficiency, we concluded that it is highly likely that the rare *DGAT1* p.L105P variant explains the gastrointestinal symptoms.

### *DGAT1* p.L105P is a partial loss-of-function allele

**Fig. 2.**
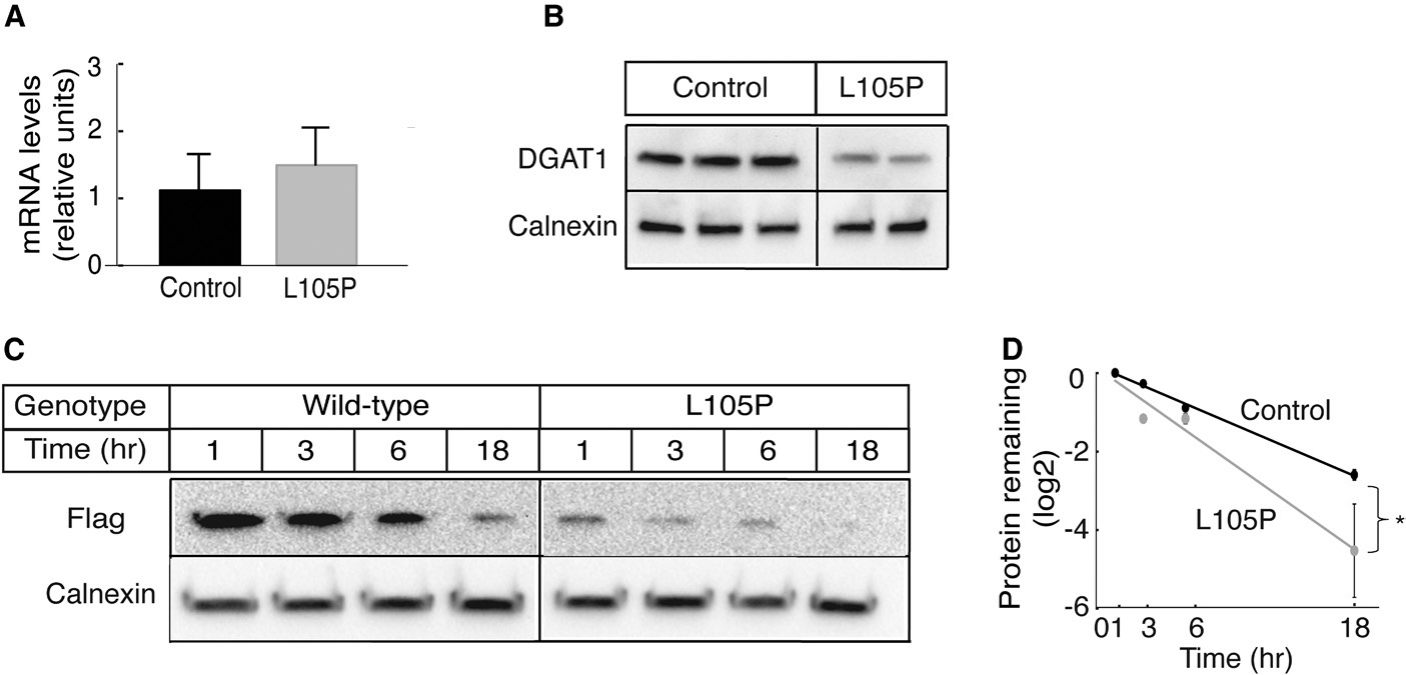
DGAT1 p.L105P protein is found at reduced levels in cells. A: *DGAT1* transcript levels are similar in control and patient fibroblasts. Relative mRNA levels determined by quantitative real-time PCR are shown here. B: Immunoblot shows less endogenous DGAT1 protein in mutant fibroblast lysate than in controls. C: FLAG-tagged wild-type and p.L105P DGAT proteins were expressed in SUM159 cells. The cells were treated with cycloheximide and lysed at different times. Immunoblot shows wild-type p.L105P DGAT1 protein may be degraded more rapidly compared with wild-type. D: Quantification of data shown in (C) and a second independent experiment. Statistical significance was determined by linear regression; *P < 0.05.

We characterized the effects of the *DGAT1* c.314C>T, p.L105P variant on the levels of DGAT1 mRNA, protein, and activity in primary dermal fibroblasts from both patients. *DGAT1* transcript levels were similar to those of control cells (**Fig. 2A**). Immunoblotting with a DGAT1-specific antisera showed reduced levels of the p.L105P protein in protein lysates (Fig. 2B). The comparable transcript, but lower DGAT1 protein levels in the mutant cells, suggested that the DGAT1 p.L105P protein is degraded more rapidly than the wild-type protein. To test for this possibility, we measured levels of expressed FLAG-tagged wild-type and p.L105P DGAT1 proteins over time in SUM159 cells after treatment with cycloheximide. The mutant p.L105P DGAT1 protein was less abundant than the wild-type at each time point and appeared to be degraded slightly faster than the wild-type (Fig. 2C, D).

**Fig. 3.**
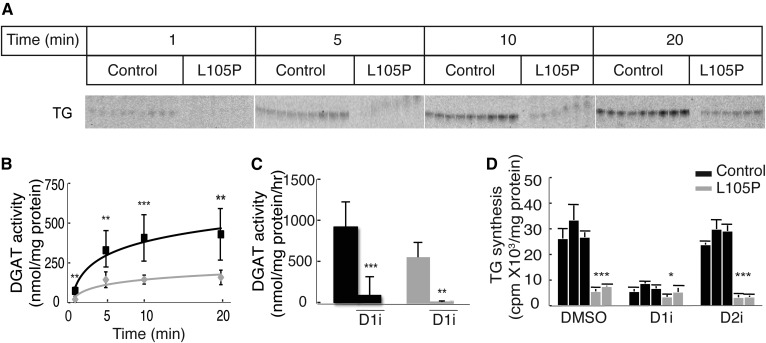
Cells with DGAT1 p.L105P enzyme have reduced, but not absent, DGAT activity. A: Lysates from DGAT1 p.L105P patient fibroblasts exhibit decreased DGAT in vitro activity. Incorporation of [^14^C]oleoyl CoA into TG in control and patient fibroblasts was measured. Lipids were separated by TLC, and the TG band for different reaction times is shown. B: Quantification of data shown in (A). Statistical significance was determined by Wilcoxon rank sum test; **P < 0.005; ***P < 0.001. C: Residual DGAT activity in patient fibroblast lysates can be further decreased by treatment with a DGAT1 inhibitor (D1i). Statistical significance was determined by Wilcoxon rank sum test; **P < 0.005; ***P < 0.001. D: TG synthesis is decreased in intact fibroblast cells of patients. The amount of [^14^C]oleate incorporated into TG in counts per minute per milligram protein is shown here. Statistical significance was determined by Wilcoxon rank sum test; ***P < 0.0001; *P < 0.05. D2i, DGAT2 inhibitor.

Analysis of in vitro DGAT1 activity in cell lysates from patient cells showed that they synthesize TGs, but at lower activity levels than those of control lysates (**Fig. 3A, B**). When corrected for reduced protein expression, the in vitro specific activity for the p.L105P protein was roughly equivalent to the activity of the wild-type protein. DGAT activity in patient lysates was decreased further by adding a DGAT1 inhibitor, demonstrating that the mutant protein has residual in vitro activity (Fig. 3C).

We also examined TG synthesis in intact cells by measuring the incorporation of [^14^C]oleate into TG after 4 h of incubation. We found that TG synthesis was markedly reduced in patient cells to levels similar to those found with treatment of control cells with a DGAT1 (but not a DGAT2) inhibitor (Fig. 3D). The discrepancy between the finding of residual activity in the in vitro assay, but not in intact cells, might relate to the V_max_ conditions employed in the in vitro assay, providing maximal access of the mutant enzyme to substrates.

**Fig. 4.**
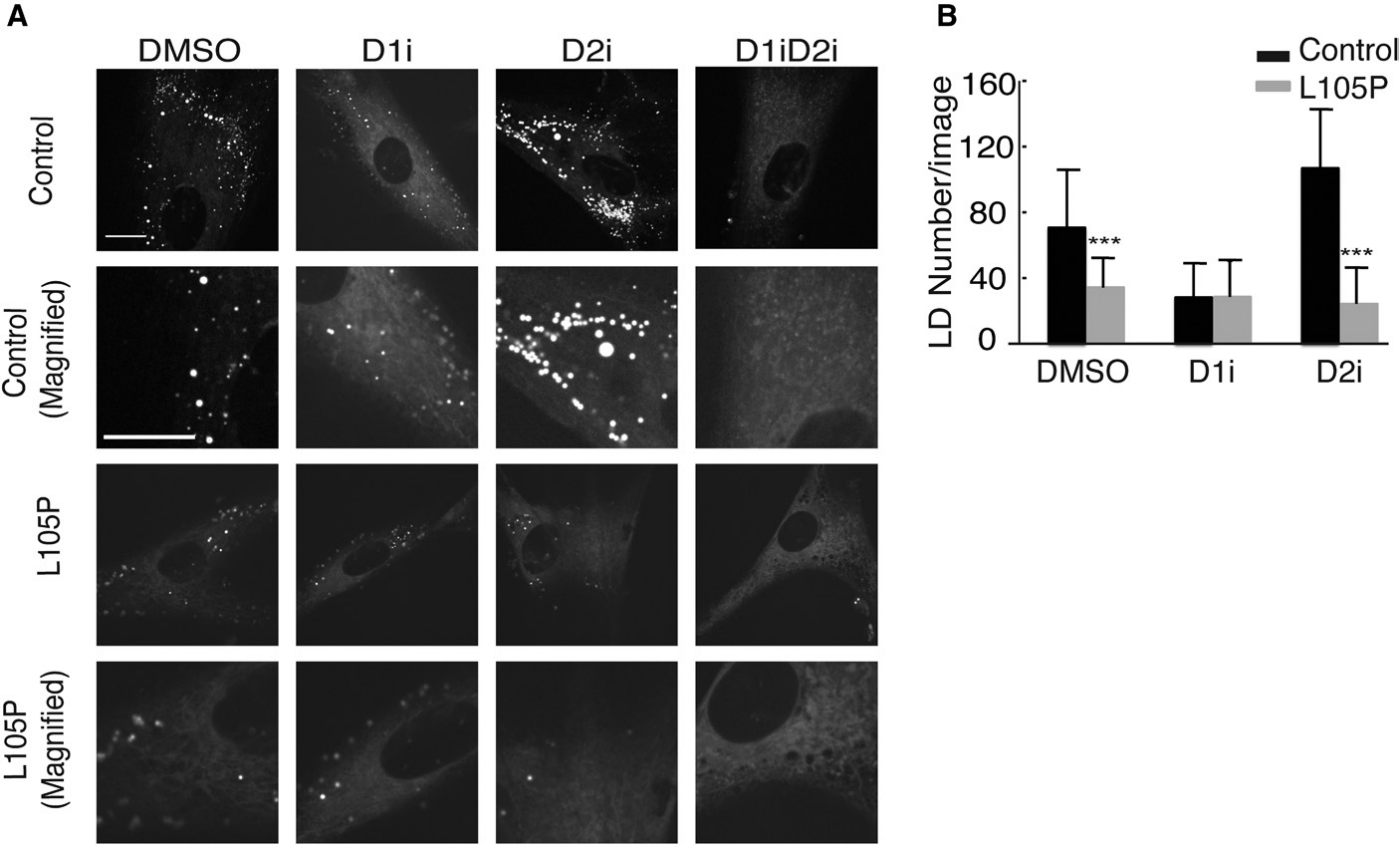
A: Mutant fibroblasts have fewer lipid droplets (LD) under DMSO and DGAT2 inhibitor (D2i) conditions after oleate treatment. Cells were stained with BODIPY 493/503 and imaged for neutral lipids. Representative images are shown. Scale bar: 30 μm. B: Lipid droplet numbers were quantified using CellProfiler. Quantification represents data from six to eight images per cell line (three controls and two patients) per condition. Statistical significance was determined by Wilcoxon rank sum test; ***P < 0.0001. D1i, DGAT1 inhibitor, D1iD2i, DGAT1 and DGAT2 inhibitors.

Because TG is stored in lipid droplets, we evaluated the effect of the *DGAT1* p.L105P mutation on cellular lipid droplets in patient fibroblasts cultured in medium containing oleate. Cells with the mutation had fewer lipid droplets after treatment with oleate under control and DGAT2-inhibited conditions. With DGAT1 inhibition, mutant and control cells had similarly reduced numbers of lipid droplets (**Fig. 4A, B**).

## DISCUSSION

Three homozygous recessive *DGAT1* mutations have now been reported that are associated with congenital diarrhea syndrome (18, 19). The first, a mutation causing deletion of exon 8, yields a complete null allele with no expressed DGAT1 protein or activity (18). More recently, two missense mutations were identified. One of these, DGAT1 p.L295P, results in less abundant DGAT1 protein than the wild-type protein, although enzymatic activity for the mutant protein was not investigated (19). Here, we describe monozygotic twin males of South Asian descent with a similar, but less severe, clinical syndrome, who were found to have a different p.L105P missense mutation in *DGAT1*. p.L105P has an allele frequency of 0.000018 (http://gnomad.broadinstitute.org), suggesting cases due to this mutation are quite rare. DGAT1 p.L105P protein levels are lower than wild-type DGAT1, but our studies reveal DGAT1 p.L105P possesses residual DGAT1 activity. Thus, in contrast to the exon 8 deletion mutation we reported previously, the p.L105P mutation appears to result in a partial loss of DGAT1 function and a less severe clinical syndrome. The differences in functional DGAT activity for these two mutations, along with the variability in clinical severity that accompanies these mutations, suggest that *DGAT1* mutations result in a spectrum of congenital diarrheal disease, with symptoms correlating with the amount of residual DGAT1 activity.

The molecular structure of DGAT1 is unknown, but the proline substitution due to the p.L105P mutation likely occurs in the first transmembrane α helix of the hydrophobic membrane-embedded enzyme (26). The N terminus of DGAT1 is localized in the cytosol and the C terminus is localized in the lumen of the endoplasmic reticulum (26). Most prediction algorithms suggest that the protein has nine transmembrane domains (TMHMM Server v. 2.0, cbs.dtu.dk/services/TMHMM/ and TOPCONS, topcons.cbr.su.se), although one study of DGAT1 topology using epitope tags found three transmembrane domains (26). Supplemental Fig. S1 shows a predicted model for DGAT1 with nine transmembrane domains and the location of currently known DGAT1 mutations. Our data from patient fibroblasts indicate that the p.L105P mutant protein is found in lower abundance than the wild-type protein. The p.L105P DGAT1 enzyme had similar activity levels as wild-type DGAT1 in vitro, but markedly reduced TG synthesis in intact cells. Our current model to explain the lower levels of the DGAT1 p.L105P protein is that some of the mutant protein is misfolded and degraded immediately after translation, with some of the remaining protein being stable and at least partially active. It is unclear why TG synthesis is so markedly reduced in intact DGAT1 p.L105P cells. One possibility is that the mutation affects substrate availability to the enzyme.

Children with DGAT1 deficiency can be very ill, and one death was reported from complications of malnutrition (18). Some patients have required rehabilitation with parenteral nutrition and intralipid, including twin A described herein. Currently, there are no therapies to increase or restore intestinal DGAT activity, so therapies are aimed at minimizing symptoms by dietary modifications. The patients reported here improved on a low-fat enteral diet, with resolution of their protein-losing enteropathy and normalization of serum immune globulin G and total protein levels. The amount of dietary fat tolerated by affected patients appears to be related to the underlying mutation and level of DGAT1 activity. For example, patients with loss of exon 8 and no DGAT1 activity tolerate approximately 4–7% of calories from fat, while patients with the p.L105P mutation tolerate up to 10% of calories from fat. Dietary fat is best tolerated by these individuals if it is given in small amounts at multiple times during the day rather than in large boluses. Patients have improved with diets of fat-free formulas or foods supplemented with measured amounts of canola oil or a mixture of sunflower and flaxseed oils. Dietary fat can be titrated to tolerance, defined by criteria of no diarrhea, normal stool α-1 antitrypsin levels, normal essential fatty acid profile, and appropriate growth for age.

Our study and our limited experience with other patients with *DGAT1* mutations provide some early guidelines for clinical management. Most importantly, these patients should be managed with a low-fat enteral diet. Patients on this regimen must be monitored for essential fatty acid deficiency, and intralipid should be administered as necessary to prevent essential fatty acid deficiencies. Monitoring should also include levels of fat-soluble vitamins, serum TGs, complete blood counts, immune globulin G and total protein levels, and stool α-1 antitrypsin levels. The latter appears to be an excellent marker for disease activity (Fig. 1). In our experience, DGAT1-deficient patients on this modified diet have shown good catch-up growth and normal development. When they are challenged with increased fat intake, they experience diarrhea, and the stool α-1 antitrypsin level increases. As clinicians become more aware of this disease, we expect that patients will be diagnosed earlier and treated successfully.

The cause of diarrhea with protein-losing enteropathy, and in most cases hypertriglyceridemia, with DGAT1 deficiency is unknown. Hypertriglyceridemia appears to be a feature of human DGAT1 deficiency (18, 19) and possibly is due to increased hepatic TG secretion mediated by DGAT2 and/or to impaired clearance of TG-rich lipoproteins. A mechanism for either is currently unclear. For the diarrhea, our current hypothesis is that DGAT1 lipid substrates from the diet accumulate at increased levels in the intestine and cause lipotoxic stress in enterocytes, resulting in cellular dysfunction. The human intestine might be particularly susceptible to the effects of DGAT1 deficiency, as human intestine appears to express *DGAT1* exclusively, whereas murine intestine expresses both *Dgat1* and *Dgat2* (18). Consistent with this, we find that DGAT1 inhibition in adipocytes during lipolysis, a condition with suppressed DGAT2 activity, exhibits signs of lipotoxicity and endoplasmic reticulum stress (Chitraju et al., unpublished observations). The mechanisms underlying the diarrhea with DGAT1 deficiency might be better investigated in a murine model lacking Dgat2 specifically in the intestine. Understanding the pathophysiology that results in diarrhea with DGAT1 deficiency may be helpful in developing treatments for affected children and possibly for making treatment of humans with DGAT1 inhibitors more tolerable.

## Supplementary Material

Supplemental Data
