# Supplementary material for: Identification and characterization of a novel DGAT1 missense mutation associated with congenital diarrhea
Source: J Lipid Res. 2017 Apr 3;58(6):1230–7. doi: 10.1194/jlr.P075119 (PMC5454518; doi:10.1194/jlr.P075119)
Supplement: Supplemental Data [file supp_58_6_1230__index.html]

Identification and characterization of a novel DGAT1 missense mutation associated with congenital diarrhea — Identification and characterization of a novel DGAT1 missense mutation associated with congenital diarrhea — Supplemental Data 

# Identification and characterization of a novel *DGAT1* missense mutation associated with congenital diarrhea

## Supplemental Data

- Supplemental Material (.pdf, 1.7 MB) - Supplemental Material
