# Supplementary material for: Identification and characterization of a novel DGAT1 missense mutation associated with congenital diarrhea
Source: J Lipid Res. 2017 Apr 3;58(6):1230–7. doi: 10.1194/jlr.P075119 (PMC5454518; doi:10.1194/jlr.P075119)
Supplement: Supplemental Data [file 10.1194_P075119_jlr.P075119-1.pdf]

Supplemental Information

Identification and characterization of a novel *DGAT1* missense mutation associated with congenital diarrhea

Nina L. Gluchowski[1,2,3], Chandramohan Chitraju[2], Joseph A. Picoraro[4], Niklas Mejhert[2], Shirly Pinto[5], Winnie Xin[3,6], Daniel S. Kamin[1,3], Harland S. Winter[3,7], Wendy K. Chung[8], Tobias C. Walther[2,3,9,10,*] and Robert V. Farese Jr.[2,3,9,*]

Division of Gastroenterology and Nutrition, Boston Children's Hospital, Boston MA 02115[1]; Department of Genetics and Complex Diseases, Harvard T.H. Chan School of Public Health, Boston, MA 02115[2]; Harvard Medical School, Boston MA 02115[3]; Division of Pediatric Gastroenterology, Hepatology and Nutrition, Columbia University Medical Center, New York, NY 10027[4]; Merck & Co., Inc., Kenilworth, NJ 07033[5]; Department of Neurology, Massachusetts General Hospital, Boston, MA 02114[6]; Division of Gastroenterology, MassGeneral Hospital for Children, Boston, MA 02114[7]; Departments of Pediatrics and Medicine, Columbia University Medical Center, New York, NY 10027[8]; Broad Institute of Harvard and MIT, Cambridge, MA 02142[9]; and Howard Hughes Medical Institute, Boston, MA 02115[10]

Supplemental Figure S1

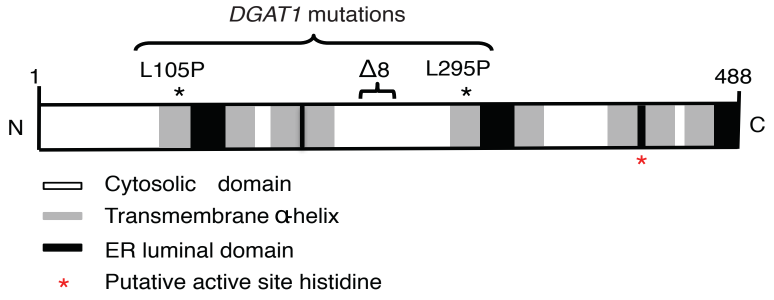

Supplemental Fig. S1. Schematic model of the DGAT1 protein showing nine predicted transmembrane domains, a histidine required for activity, and the location of amino acid changes due to the three human *DGAT1* mutations. Adapted from adapted from TMHMM Server v. 2.0 and McFie et al 2010. Δ8: chromosome 8 145541756 A->G.
